# Back loading estimation during team handling: Is the use of only motion data sufficient?

**DOI:** 10.1371/journal.pone.0244405

**Published:** 2020-12-22

**Authors:** Antoine Muller, Philippe Corbeil

**Affiliations:** 1 Institut de Recherche Robert-Sauvé en Santé et en Sécurité du Travail (IRSST), Montréal, QC, Canada; 2 Univ Lyon, Univ Gustave Eiffel, Université Claude Bernard Lyon 1, LBMC UMR_T 9406, Lyon, France; 3 Department of Kinesiology, Faculty of Medicine, Université Laval, Quebec City, QC, Canada; 4 Centre for Interdisciplinary Research in Rehabilitation and Social Integration (CIRRIS), Centre Intégré Universitaire de Santé et de Services Sociaux de la Capitale-Nationale (CIUSSS-CN), Quebec City, QC, Canada; University of Rome, ITALY

## Abstract

Analyzing back loading during team manual handling tasks requires the measurement of external contacts and is thus limited to standardized tasks. This paper evaluates the possibility of estimating L5/S1 joint moments based solely on motion data. Ten subjects constituted five two-person teams and handling tasks were analyzed with four different box configurations. Three prediction methods for estimating L5/S1 joint moments were evaluated by comparing them to a gold standard using force platforms: one used only motion data, another used motion data and the traction/compression force applied to the box and one used motion data and the ground reaction forces of one team member. The three prediction methods were based on a contact model with an optimization-based method. Using only motion data did not allow an accurate estimate due to the traction/compression force applied by each team member, which affected L5/S1 joint moments. Back loading can be estimated using motion data and the measurement of the traction/compression force with relatively small errors, comparable to the uncertainty levels reported in other studies. The traction/compression force can be obtained directly with a force measurement unit built into the object to be moved or indirectly by using force platforms on which one of the two handlers stands during the handling task. The use of the proposed prediction methods allows team manual handling tasks to be analyzed in various realistic contexts, with team members who have different anthropometric measurements and with different box characteristics.

## Introduction

Team handling occurs when two or more people are involved in a manual handling task. Team handling is common in many occupations, such as the military [1], the medical field for patient transfers [2–7], the construction industry [8, 9], and by movers [10]. The team movement strategy emerges from the interaction of the task, the environment, and the individuals. Particular task constraints, such as handling a heavy or bulky load, often affect team handling. Other constraints may emerge from the environmental setting, imposed by either the work organization or a constrained space.

Biomechanical studies have been conducted to analyze team handling. Some of them have involved kinematic analysis during patient transfers [2, 11] using the Lumbar Motion Monitor [12]. Other studies, conducted in the laboratory, have investigated low back loading [8, 9, 13, 14]. Traditionally, this type of study requires the measurement of the handler’s kinematics and of external force contacts: either ground reaction forces (GRF) using force platforms or hand forces (HF) using instrumented handles. The use of force sensors limits the realism of experimental tasks by obliging handlers to use handles or constraining their foot displacements [13]. Subjects must either remain on a platform throughout the handling task [9, 14] or follow a predefined footstep pattern on several platforms [8]. These experimental aspects limit the ecological validity of the analyses performed [13].

Yet, as Barrett and Dennis [13] stated in their review, “there is a need to perform studies that examine effort and load among team members.” Team handling increases the lifting capacity compared to individual handling and is commonly recommended to reduce the load during manual handling of heavy loads; despite its benefits, though, some factors represent a risk for low back injuries [13]. Forces and loads are distributed differently on the musculoskeletal system in individual vs. team handling. In addition, the risk of injury to an individual who is carrying a larger proportion of the load can increase. This risk may be exacerbated when team members have unequal strength and stature. The risk may also increase when the movement strategy is not adapted to the context due to differences in intention, motor control (refined by work experience), lack of synchrony, etc.

Recently, prediction methods have been developed to estimate contact forces based only on motion data [15, 16] and applied to individual handling tasks [17–19]. These methods use dynamic equations and are based on a contact model and an optimization-based method. However, team handling involves additional issues. The distribution of forces applied to the box between the team members is an essential element to be considered. Moreover, both team members can exert additional effort on the box, for example to increase stability or due to a lack of synchrony. These efforts influence back loading [20] and therefore need to be taken into account.

The aim of this paper was to evaluate whether it is possible to use only motion data to accurately estimate back loading during team handling. If these data are not sufficient, what additional data must be considered? For this purpose, tandem handling tasks were analyzed in the laboratory. Three different prediction methods were used to estimate back loading: one used only motion data, another used motion data and the traction/compression force applied to the box and the third used motion data and the ground reaction forces of one team member. The evaluation consisted in, first, comparing the predicted and measured GRF and, second, comparing the L5/S1 joint moments computed with the predicted and measured GRF.

## Materials and methods

### Experimental procedure

Ten subjects (6 males, 4 females, age: 22 ± 1 years old, height: 176 ± 8 cm, mass: 72 ± 7 kg) participated in the experiment. The study was approved by the local institution’s Research Ethics Committee and each subject signed an informed consent form prior to the experiment. Subjects were young university students with very little manual material handling experience. They had had no injuries in the last six months. Five two-person teams were created, including mixed, women’s and men’s teams, with various height differences between members of each team (Table 1).

**Table 1:** Individual data on each team.

	Team member ♯1			Team member ♯2		
	Height [cm]	Mass [kg]	Sex	Height [cm]	Mass [kg]	Sex
Team 1	165	59	F	169	63	F
Team 2	173	69	M	171	68	F
Team 3	178	69	M	191	86	M
Team 4	172	65	F	179	83	M
Team 5	186	74	M	179	82	M

**Fig 1:**
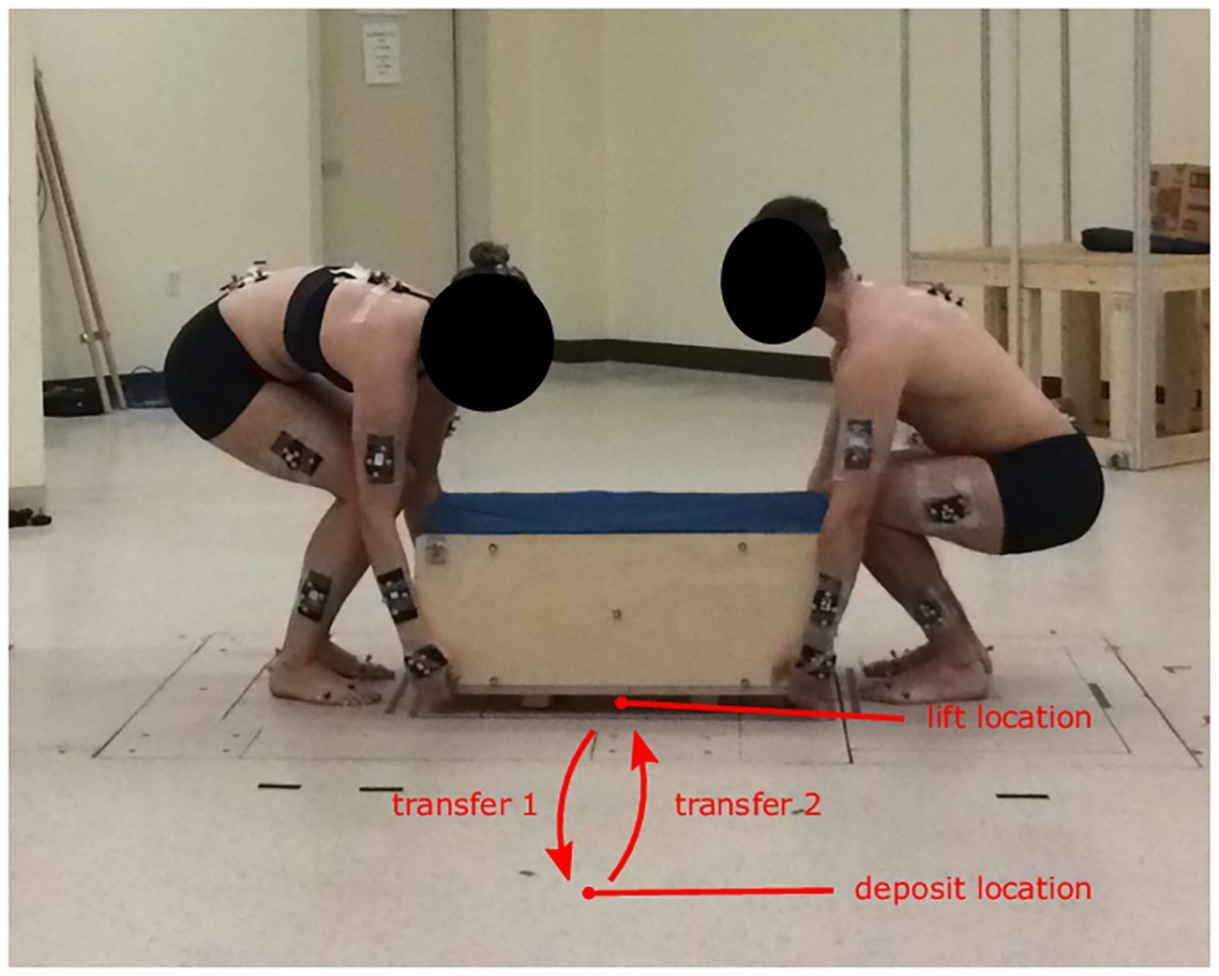
Experimental protocol with two team members lifting the box at the beginning of transfer 1.

Each trial was composed of two handling tasks (Fig 1): transfer a box from a lift location centered in relation to their body (facing the load to be lifted) to a deposit location 36 cm off-centered to the right of one team member and to the left of the other, and then bring it back again. Both lift and deposit locations were 4 cm above the ground and were marked with tape. Each team member began the trial in a neutral upright standing position with arms on each side of the body. After the experimenter gave the start signal, team member ♯1 was instructed to signal the other member of the tandem to begin the lift. After the first deposit, a short pause of approximately 2 seconds was taken in the neutral upright position without any load in their hands. Then team member ♯1 gave the signal to start the second box transfer (return). After the task, participants were asked to stand in a neutral position. Each handling trial thus comprised 9 successive phases: standing in a neutral position without a load in the hands; pre-maneuver 1 (starting at the first touch on the box and ending at the instant of lift); transfer 1; post-maneuver 1 (beginning at the instant of deposit and ending when the participants were no longer touching the box); pause; pre-maneuver 2; transfer 2; post-maneuver 2; and standing in a neutral position without a load in the hands.

One box was adapted to have four different configurations: *B*17_*E*_: 17 kg evenly distributed; *B*51_*E*_: 51 kg evenly distributed; *B*51_♯1_: 51 kg where the center of mass had been shifted toward team member ♯1; and *B*51_♯2_: 51 kg where the center of mass had been shifted toward team member ♯2. Several weights were added to a rigid base structure to obtain the *B*51_*E*_, *B*51_♯1_ and *B*51_♯2_ configurations. For each one, the same weights were added to ensure the same total mass. The box’s dimensions were 80.5 × 44.5 × 32 cm. Four repetitions of each tasks (including an outward and a return phase) were performed back to back with each configuration. Subjects were given a two-minute familiarization period to practice box transfers with configuration *B*51_*E*_. After this familiarization period, trials with the *B*17_*E*_ configuration were performed. Then, the order of *B*51_*E*_, *B*51_♯1_ and *B*51_♯2_ was randomized. Weight changes between conditions were executed out of sight of the participants.

**Fig 2:**
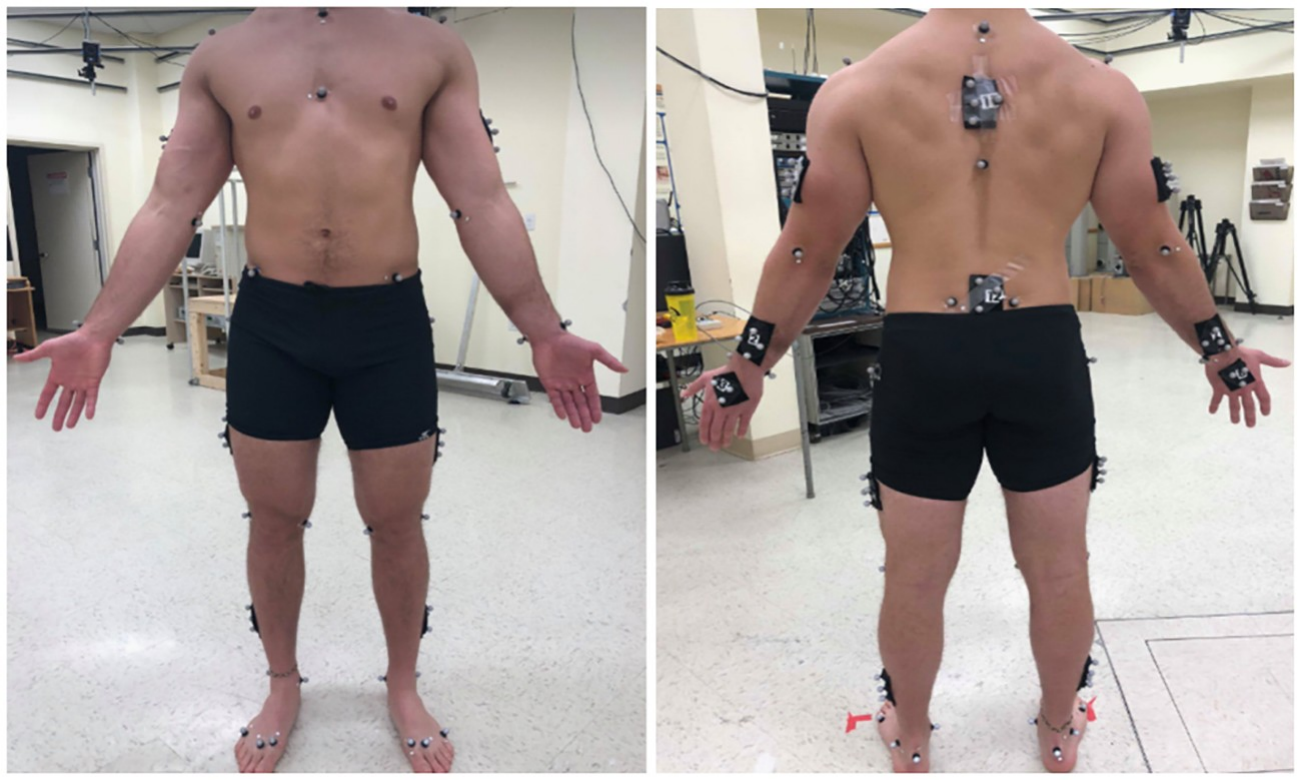
Experimental markers initially located on each participant.

A motion capture system (10 cameras, Vicon, Peak, UK) recorded at 100 Hz the 3D coordinates of markers located on both participants (see S1 File) and on the box. At the beginning of each experiment, the locations of 82 markers on each participant were recorded (Fig 2). Some of them were located on anatomical landmarks following the ISB recommendations [21, 22] and the others constituted clusters. Fifty-eight markers were left on each participant during handling trials. Markers on the participants were used to estimate anatomical landmarks position during tasks, then used as input to assess joint coordinates (more details about this step are provided under “Inverse kinematics”). In addition, 10 markers were located on the box. Four of them were used to construct a coordinate system associated with the box and the others were used to reconstruct these 4 markers in case of occlusion.

GRF were measured at 2000 Hz using three force platforms (Advanced Mechanical Technology Inc., Watertown, MA). One subject stood on one of the force platforms and the second subject stood on two, with one foot on each platform. Subjects were instructed not to move their feet during the box transfers.

### Biomechanical model

Back loading was estimated based on a whole-body osteoarticular model, composed of 16 rigid segments (pelvis, lower trunk, upper trunk, head, upper arms, lower arms, hands, upper legs, lower legs and feet) linked by 15 joints corresponding to 35 degrees of freedom (3 for the pelvis / lower trunk joint, 3 for the lower trunk / upper trunk joint, 3 for the neck, 3 for each shoulder, 2 for each elbow, 2 for each wrist, 3 for each hip, 1 for each knee and 2 for each ankle). The geometrical parameters were subject-specific, calibrated using motion capture data and an optimization-based method [23–25]. Body segment inertial parameters (BSIP) were extracted from anthropometric tables [26].

For the prediction methods, discrete contact points were defined on the model, corresponding to the potential contact points with the ground or with the box. Fourteen points under each foot and 11 points under each hand were defined to map the contact area [15, 16].

### Inverse kinematics

From the positions of 36 anatomical landmarks (estimated from the positions of the rigid clusters), the joint coordinates were computed with a multibody optimization [27] and then filtered with a 4th-order Butterworth low-pass filter with a cut-off frequency of 6 Hz and no phase shift [28].

### External forces

**Fig 3:**
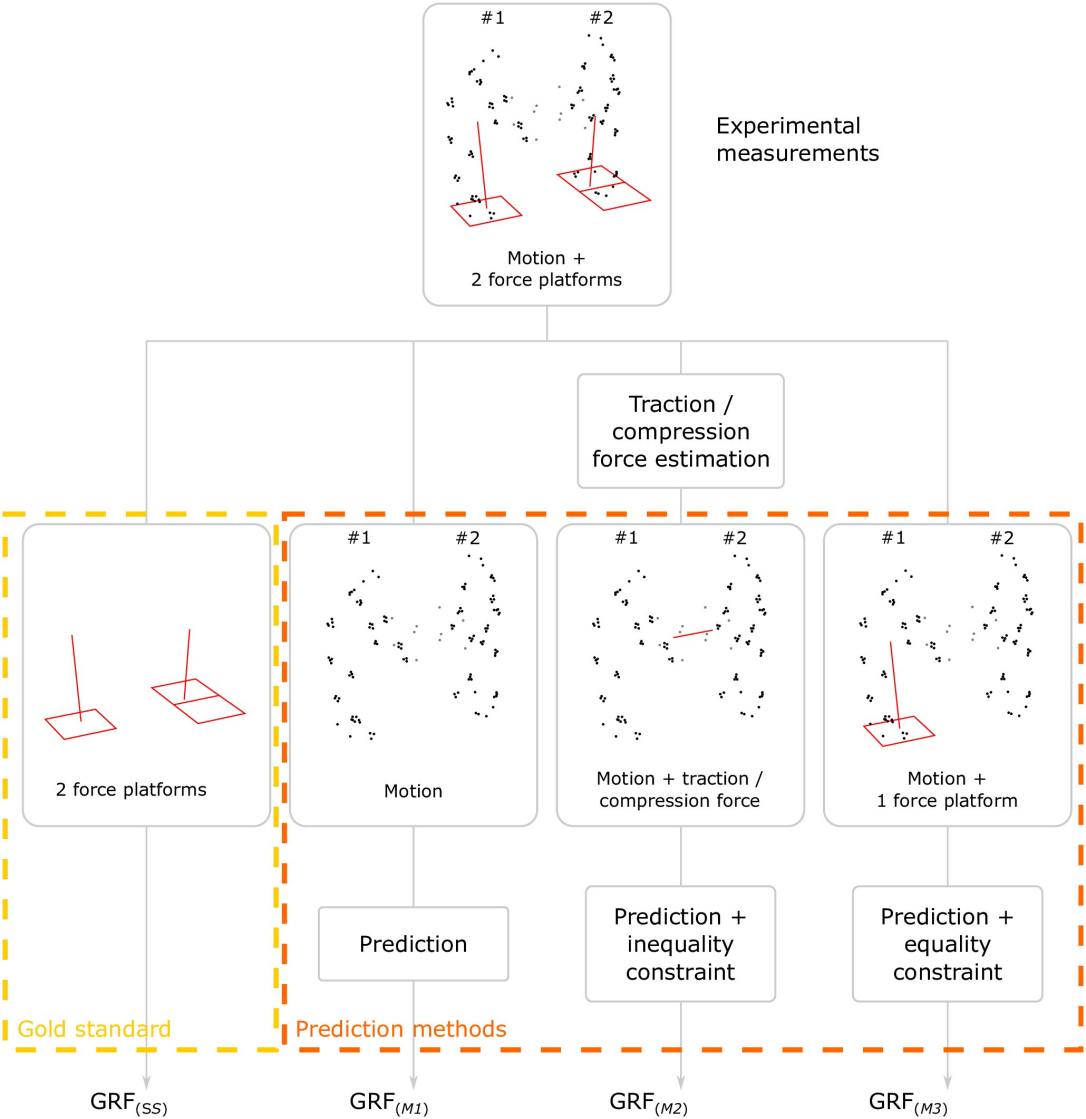
Setup of the gold standard (*GS*) and the three prediction methods (*M1*, *M2*, and *M3*) used to estimate GRF.

GRF were estimated by three different prediction methods (*M1*, *M2* and *M3*), each compared to force platform measurement, considered as the gold standard (Fig 3). The three prediction methods differed in the type of experimental input data used: *M1* used only motion data, *M2* used motion data and the traction/compression force applied to the box and *M3* used motion data and one team member’s GRF.

**Fig 4:**
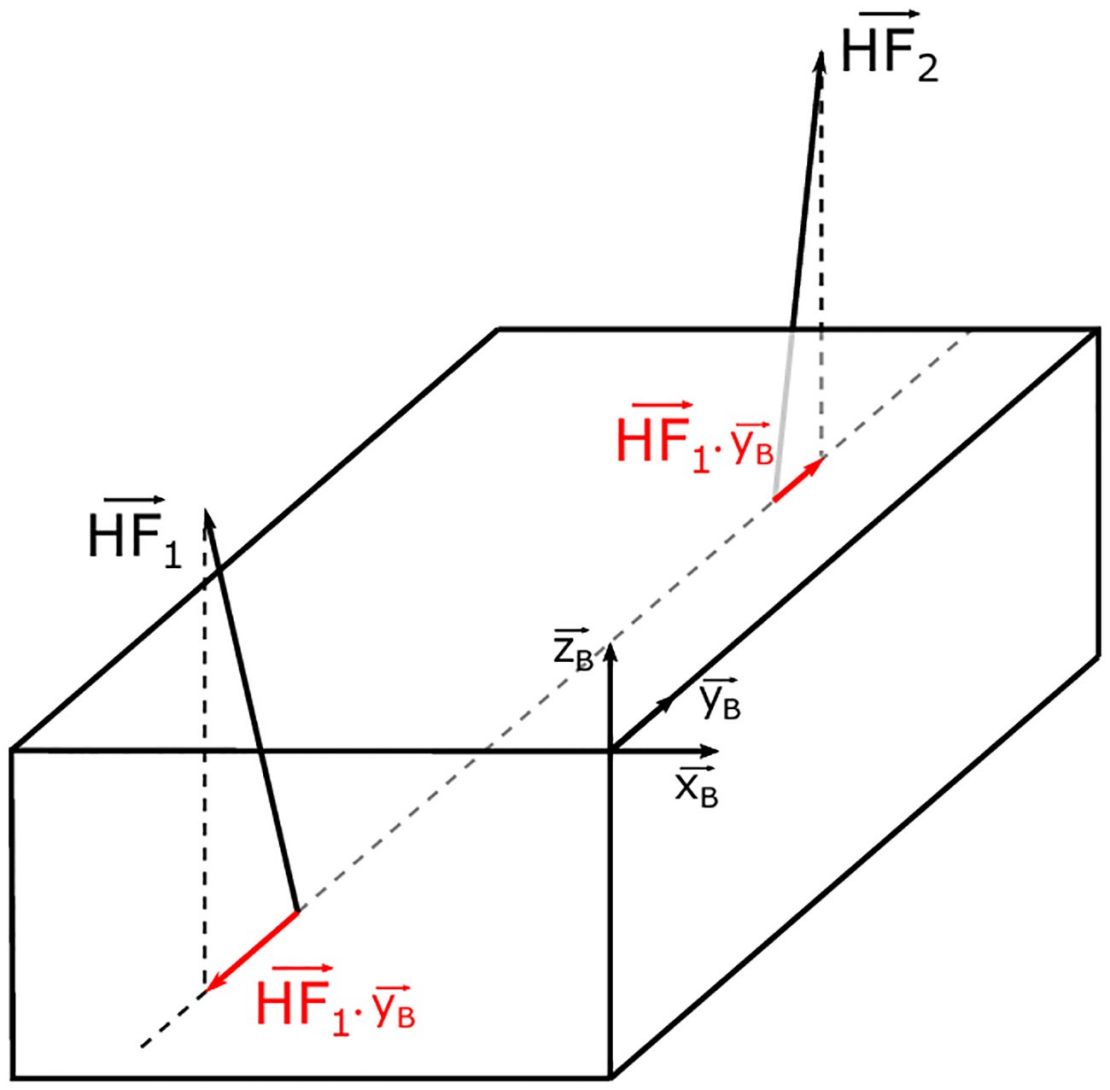
HF applied by both team members and their respective projections on the longitudinal axis of the box $\vec{y_{B}}$.

As the traction/compression force was not measured during these experiments, this force was calculated from the HF estimates. HF applied by each team member were estimated from the GRF and motion measured, by subtracting each body segment’s contribution to GRF [29]. The traction/compression force represents the minimum common HF applied by both team members in the longitudinal direction of the box (Eq 1) (Fig 4). The longitudinal direction was estimated using the box markers. At each sample time, it corresponded to a scalar value.

Considering that the amplitude of the traction/compression force applied by each team member was equal and opposite, no resultant acceleration of the box emerged due to these forces. The acceleration of the box was directly proportional to the HF applied on the box by both team members along the three orthogonal directions. This point is discussed further under “Limits and perspectives”.

$$\begin{array}{c} F_{TC}=\text{min}\left(\vec{HF_{1}}.\vec{y_{B}},\;\vec{HF_{2}}.\vec{y_{B}}\right) \end{array}$$ (1)

$\vec{HF_{1}}$ and $\vec{HF_{2}}$ are the HF applied by team member ♯1 and team member ♯2, respectively. $\vec{y_{B}}$ represents the longitudinal axis of the box.

The rationale behind the addition of the traction/compression force applied to the box (Method *M2*) is that this is a component whose effect on the box cannot be captured from the whole-body and box kinematics. Thus, it is important information to ensure a good estimate of back loading.

The next three subsections describe the gold standard and the three prediction methods.

#### Gold standard

The gold standard method consisted in using the measurements produced by the force platforms directly. Since only the L5/S1 joint moment was computed, only the resultant GRF for each subject was considered. The two platforms under one of the two participants were used here only to measure the resultant GRF.

#### Prediction method *M1*

Prediction method *M1* used a motion-based prediction method developed earlier [17, 18]. Only the kinematics of the two subjects and the box were used as input data for method *M1* to predict L5/S1 joint moment. The algorithm applied a contact model with an optimization-based method. Each contact point defined on the biomechanical models was a potential linear force. The estimated contact forces were the minimum forces in a least-squares sense while ensuring that the dynamics equations applied to both team members and the box. To improve the numerical resolution of the algorithm, the three systems (team members and box) were solved separately.

The box’s mass was obtained by weighing it with a force platform. The position of its center of mass was estimated by placing the box on a force platform with two different orientations. Considering the low angular accelerations of the box and its complex geometry (i.e., the distribution of masses), the box’s inertia was not taken into consideration.

#### Prediction method *M2*

Method *M2* used motion data (kinematics of both subjects and box) and the traction/compression force input applied to the box. As described before, traction/compression force was not measured but estimated. It indicated that each team member applied at least this force in the longitudinal direction of the box. Prediction method *M2* used a similar algorithm to *M1*. An additional inequality constraint was added in the optimization procedure: the HF applied by each team member in the longitudinal direction of the box must be greater than or equal to the measured traction/compression force.

#### Prediction method *M3*

Method *M3* used motion data from one team member and the other team member’s GRF to estimate the L5/S1 joint moments of the first team member. The idea underlying *M3* was to estimate the error associated with the use of a single force platform, which may be the situation for many potential users of this method. It is important to understand that, when using team member ♯1’s motion data and GRF, the estimation of team member ♯1’s L5/S1 joint moments is equivalent to using the gold standard method: the error is therefore zero; the same is true for team member ♯2. For the other user (the one who is not standing on a force platform), the L5/S1 joint moment estimates depend on input data provided by the subject’s motion and the other team member’s GRF.

Prediction method *M3* was based on the same algorithm as for *M1*. An additional equality constraint was added in the optimization procedure: the GRF of one team member must be equal to those measured with the force platforms.

### L5/S1 joint moments

L5/S1 joint moments were estimated with a recursive Newton-Euler algorithm [30], using a bottom-up approach. Measured or predicted GRF were used depending the method in question.

To estimate back loading during team handling, marker-based motion capture and GRF measurements are the most widely used methods in previous studies [31–33] and will be referred to as the gold standard in this study.

### Analysis software

All prediction methods and L5/S1 joint moment estimation methods were implemented and processed with CusToM (Customizable Toolbox for Musculoskeletal simulation [34]). CusToM is a toolbox developed in Matlab^®^ that enables musculoskeletal analysis based on inverse dynamics approaches with a high level of customization.

### Data analysis

Prediction methods were evaluated by comparing the predicted and measured GRF and the L5/S1 joint moments computed with the predicted and measured GRF during the transfer phases. The asymmetrical moment was defined as the vector sum of the lateral bending and torsion moment components. For each comparison, a Pearson correlation coefficient, a root mean square error (RMSE) and a relative RMSE (rRMSE) (Eq 2) [35] were computed. For the L5/S1 joint moments, the mean error was also computed. A positive value corresponded to an underestimate and a negative value corresponded to an overestimate.

$$\begin{array}{c} rRMSE=\frac{RMSE}{\frac{1}{2}\sum_{i=1}^{2}(\underset{t\in [0,T]}{\text{max}}\left(u_{i}\left(t\right)\right)-\underset{t\in [0,T]}{\text{min}}\left(u_{i}\left(t\right)\right))} \end{array}$$ (2)

*u*_1_ and *u*_2_ correspond to the predicted and measured data, respectively, and instant *t* varies from 0 to *T* (here *t* varies between the lift and deposit instants).

A two-way repeated measures ANOVA was conducted on the Pearson correlation coefficient, the RMSE and the rRMSE by combining the three prediction methods (*M1*, *M2* and *M3*) and the four box configurations (*B*17_*E*_, *B*51_*E*_, *B*51_♯1_ and *B*51_♯2_).

Method *M3* can be used in two different ways: either using team member ♯1’s GRF or using team member ♯2’s GRF. Analyzing team member ♯1 using team member ♯1’s GRF is equivalent to using the gold standard method. Analyzing team member ♯2 using team member ♯2’s GRF is also equivalent to using the gold standard method. Thus, method *M3* was evaluated by analyzing team member ♯1 using team member ♯2’s GRF and team member ♯2 using team member ♯1’s GRF. Comparison measures of each repetitive trial were averaged. Significant effects were analyzed using a Bonferroni post hoc test. The sphericity assumption was tested with Mauchly’s test and, when this assumption was violated, the Huynh–Feldt correction was applied. The significance level was set a priori at 0.05. All statistical analyses were performed with IBM SPSS^®^ software (version 26.0).

## Results

Tables 2 and 3 show the comparison measures for the three prediction methods and the four box configurations, respectively. Statistical significance (main and interaction effects) is described in the S1 File.

**Table 2:** Comparison between measured and predicted GRF and L5/S1 joint moments computed with predicted and measured GRF for the three prediction methods.

			*M1*	*M2*	*M3*	Main effect (p-value)
GRF	V	r	0.99	0.99	0.99	-
		RMSE [N/kg]	0.09	0.09	0.10	-
		rRMSE [%]	1.8	1.8	2.0	-
	AP	r	0.31	0.90	0.90	*M1* < *M2*, *M3* (< 0.001)
		RMSE [N/kg]	0.50	0.09	0.06	*M1* > *M2* > *M3* (< 0.001)
		rRMSE [%]	54	8.2	6.0	*M1* > *M2*, *M3* (< 0.001)
	ML	r	0.84	0.84	0.84	-
		RMSE [N/kg]	0.09	0.09	0.07	-
		rRMSE [%]	10	9.7	7.8	*M1* > *M3* (0.024)
L5/S1 joint moments	S	r	0.98	0.98	0.96	-
		RMSE [Nm]	29	11	16	*M1* > *M3* > *M2* (< 0.001)
		rRMSE [%]	12	5.3	7.1	*M2* < *M1*, *M3* (0.001)
	A	r	0.64	0.65	0.63	-
		RMSE [Nm]	10	9.3	14	*M2* < *M1*, *M3* (0.008)
		rRMSE [%]	15	14	18	*M1* > *M2* (0.027)

V: vertical; AP: antero-posterior; ML: medio-lateral; S: sagittal; A: asymmetrical.

**Table 3:** Comparison between measured and predicted GRF and L5/S1 joint moments computed with predicted and measured GRF for the four different box configurations.

			*B*17_*E*_	*B*51_*E*_	*B*51_♯1_	*B*51_♯2_	Main effect (p-value)
GRF	V	r	0.99	0.99	0.98	0.99	-
		RMSE [N/kg]	0.10	0.10	0.09	0.09	-
		rRMSE [%]	2.2	1.8	1.7	1.8	-
	AP	r	0.71	0.68	0.71	0.71	-
		RMSE [N/kg]	0.09	0.27	0.26	0.25	*B*17_*E*_ < *B*51_*E*_, *B*51_♯1_, *B*51_♯2_ (< 0.001)
		rRMSE [%]	14	25	26	25	*B*17_*E*_ < *B*51_*E*_, *B*51_♯1_, *B*51_♯2_ (< 0.001)
	ML	r	0.83	0.84	0.83	0.85	-
		RMSE [N/kg]	0.06	0.09	0.08	0.08	*B*17_*E*_ < *B*51_*E*_, *B*51_♯1_ (0.011)
		rRMSE [%]	8.9	9.6	9.1	9.3	-
L5/S1 joint moments	S	r	0.98	0.97	0.98	0.98	-
		RMSE [Nm]	14	21	19	22	*B*17_*E*_ < *B*51_*E*_, *B*51_♯1_, *B*51_♯2_ (< 0.001)
		rRMSE [%]	7.7	8.4	7.4	9.2	-
	A	r	0.51	0.68	0.73	0.64	*B*17_*E*_ < *B*51_*E*_, *B*51_♯1_, *B*51_♯2_ (0.026)
		RMSE [Nm]	8.7	12	12	12	*B*17_*E*_ < *B*51_*E*_, *B*51_♯1_, *B*51_♯2_ (0.015)
		rRMSE [%]	16	16	14	17	-

V: vertical; AP: antero-posterior; ML: medio-lateral; S: sagittal; A: asymmetrical.

### GRF estimation

For the vertical axis, no significant differences in any of the dependent variables were observed between Methods or Box configurations. The correlations were 0.99 and the errors approximately 2%. The vertical axis contained the greatest forces and amplitudes since it contained the weights of the subjects and the box.

**Fig 5:**
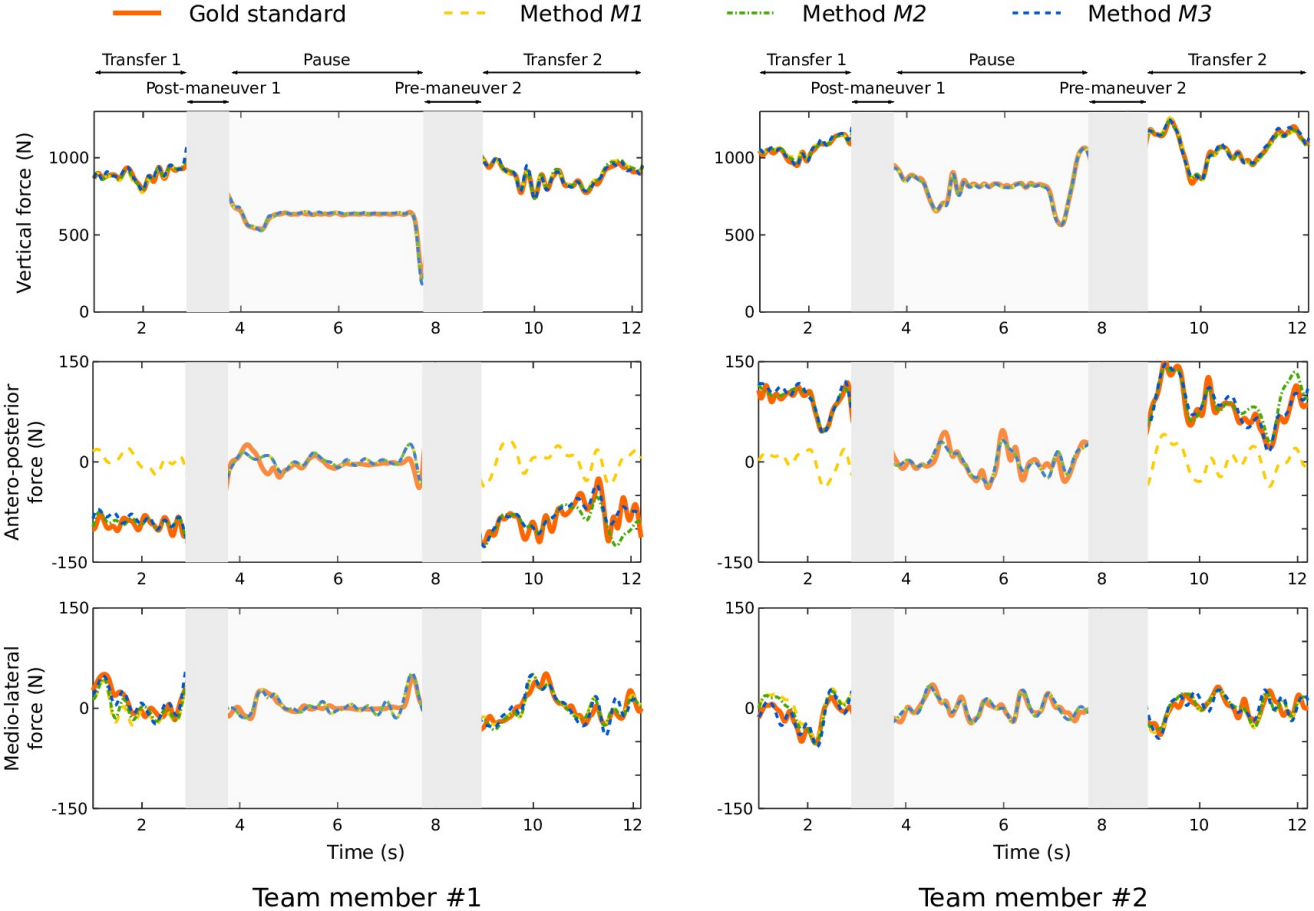
Representative example of predicted and measured GRF. The three graphs on the left represent team member ♯1’s GRF and the three graphs on the right represent team member ♯2’s GRF. The orange curves represent the gold standard, the yellow curves *M1*, the green curves *M2* and the blue curves *M3*. The different areas of the trial are highlighted: the handling phases are the ones with no background shading (areas on the left and on the right of each graph); these areas were taken into account in the statistics. The central area represents the break between two handling tasks. The phases shaded in gray are manipulation phases (pre-grip and post-deposit); these phases were not taken into account by the prediction methods.

**Fig 6:**
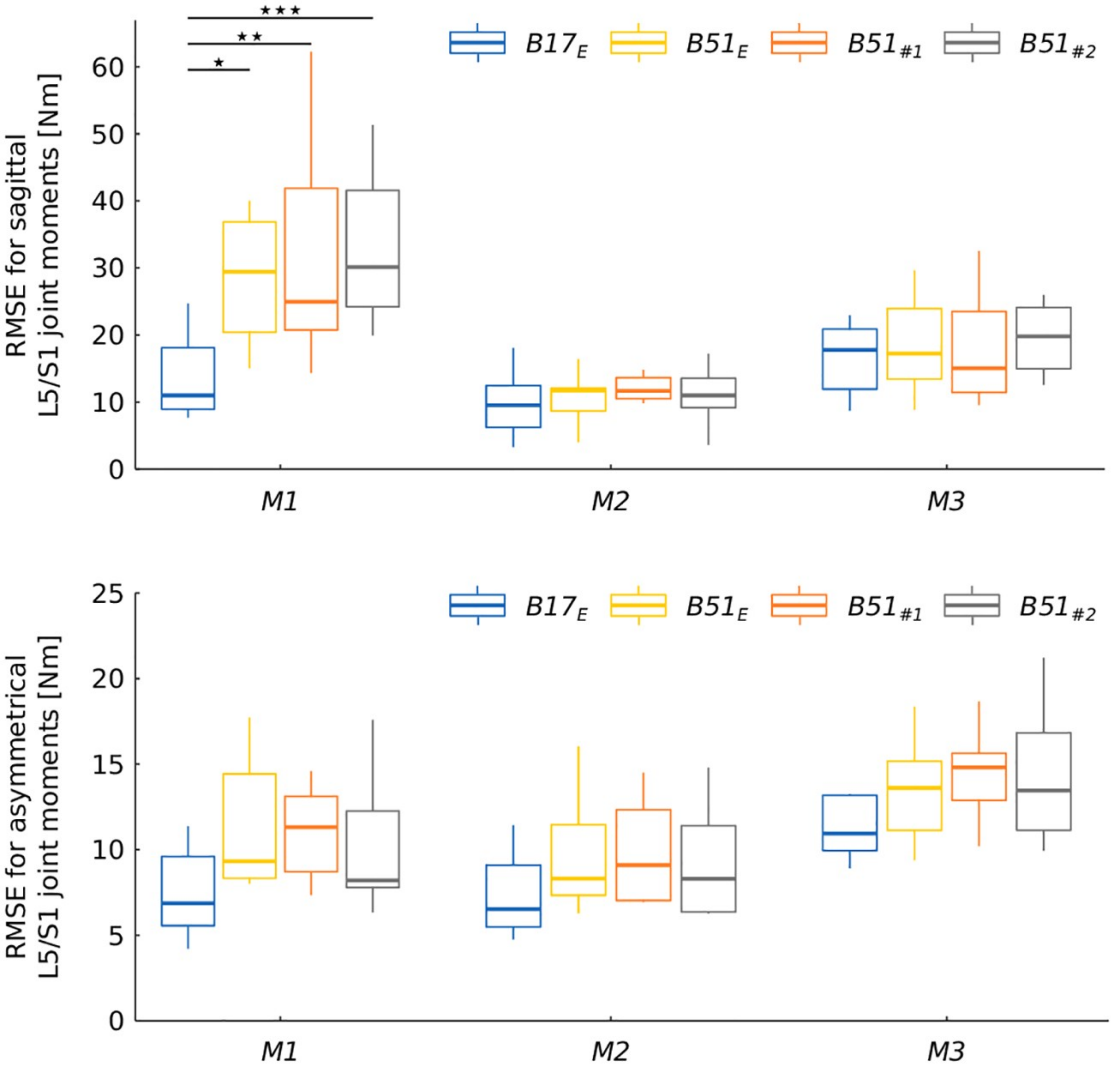
RMSE for sagittal and asymmetrical L5/S1 joint moments according to prediction method and box configuration. * significance level ≤ 0.05; ** significance level ≤ 0.01; *** significance level ≤ 0.001.

Significant differences appeared for the antero-posterior axis. *M1* had a lower correlation (mean: 0.31) and higher errors (mean: 54%) than the other methods. In the antero-posterior axis, the predicted forces were close to zero with *M1*, whereas each team member applied a non-zero opposite force (Fig 5). This force was considered in *M2* and *M3*, resulting in a mean correlation of 0.90 with a mean error of less than 10%. There were fewer errors for *B*17_*E*_ than the other three box configurations (mean: 0.09 N/kg vs. approximately 0.25 N/kg for the other axes). Moreover, a significant interaction (p ≤ 0.001) was found between both factors for the antero-posterior axis (Fig 6). With *M1*, a lower error level was obtained with *B*17_*E*_ than the other box types, while no significant difference between box configurations was observed for the other two methods.

For the medio-lateral axis, few significant differences were observed between Methods or Box configurations (Tables 2 and 3); the mean correlation was about 0.84 with mean errors less than or equal to 10%. The medio-lateral axis was the axis with the lowest force amplitudes.

### L5/S1 joint moment estimation

Significant differences in RMSE and rRMSE were observed for the sagittal axis. RMSE were the highest using *M1* and the lowest with *M2*, where the mean error was 11 Nm. rRMSE were lower using *M2* than with the other two methods. In addition, the RMSE for *B*17_*E*_ was lower than for the other three box configurations. Moreover, as with GRF in the antero-posterior axis, a significant interaction between Method and Box configuration (p ≤ 0.001) was found for the sagittal axis. With *M1*, a lower error level was obtained with *B*17_*E*_ than with the other box types, while the other two methods produced no significant difference between box configurations.

For the asymmetrical component, few significant differences were observed between methods (Tables 2 and 3). Since RMSE values were lower and rRMSE values higher, amplitudes were lower for the asymmetrical component than for the sagittal axis.

For the sagittal moment, the mean error was -27 Nm, -3.7 Nm, and 0.82 Nm for methods *M1*, *M2* and *M3*, respectively. For the asymmetrical moment, the mean error was -3.9 Nm, -2.0 Nm, and -10 Nm for methods *M1*, *M2* and *M3*, respectively.

Errors concerning sagittal and asymmetrical back loadings for each team member are reported in Table 4 for the sagittal axis. RMSE ranged between 16 and 52 Nm for *M1*, 4 and 19 Nm for *M2* and 11 and 27 Nm for *M3*.

**Table 4:** RMSE for sagittal (S) and asymmetrical (A) L5/S1 joint moments for each team member and each prediction method.

RMSE [Nm]		Team member ♯1			Team member ♯2			Team (mean)		
		*M1*	*M2*	*M3*	*M1*	*M2*	*M3*	*M1*	*M2*	*M3*
Team 1	S	29	11	11	39	19	11	34	15	11
	A	8.4	8.1	19	12	13	14	10	11	17
Team 2	S	16	10	19	52	16	27	34	13	23
	A	7.1	6.7	13	11	9.3	11	9.0	8.0	12
Team 3	S	22	4.2	15	39	12	25	30	8.2	20
	A	7.3	6.1	14	12	12	14	9.9	9.1	14
Team 4	S	29	11	20	26	11	13	27	11	16
	A	10	9.5	20	15	13	15	12	11	17
Team 5	S	19	9.5	14	16	10	11	18	10	12
	A	8.1	7.0	10	8.4	7.6	11	8.2	7.3	11

## Discussion

Three prediction methods were compared to a gold standard method using force platforms. The errors in GRF and L5/S1 joint moment estimation were analyzed.

### GRF estimation

The greatest difference between the prediction methods appeared on the antero-posterior axis, where *M1* produced a much larger error than the other methods. This method, which considers only motion data, predicts only forces required to generate the box movement and not the traction/compression force applied by team members. This force can be applied to increase stability; it may reflect a lack of synchrony between the two handlers or may be applied voluntarily to decrease low back moments [20]. Because this force does not directly influence the motion of the box, it is necessary to acquire additional information to improve the accuracy of the estimate. The traction/compression force was used directly as an input in *M2* and indirectly via the GRF of one of the team members for *M3* and thus greatly reduced prediction errors in the antero-posterior direction. A recent study reported a similar problem when estimating medio-lateral force underneath each foot based solely on motion data [18].

For similar handling tasks performed individually, Larsen et al. [19] reported RMSE of 0.44 N/kg, 0.55 N/kg and 0.21 N/kg and Muller et al. [18] reported RMSE of 0.24 N/kg, 0.08 N/kg and 0.40 N/kg for the vertical, antero-posterior and medio-lateral GRF, respectively. These reported errors were the mean errors for each foot and therefore included the errors made in the distribution of forces between both feet. For this reason, these values are not directly comparable to those reported in this paper. However, when the traction/compression force is considered (*M2* and *M3*), the errors are of the same order of magnitude as those reported in the literature for individual handling. The distribution of forces between team members is therefore well accommodated by the prediction methods, regardless of the box’s mass and the mass distribution.

### L5/S1 joint moment estimation

The largest errors obtained with *M1* for the sagittal L5/S1 joint moments came directly from the unconsidered antero-posterior forces. This error is particularly large for box configurations *B*51_*E*_, *B*51_♯1_ and *B*51_♯2_, corresponding to the heaviest masses. The team members applied a higher traction force for these boxes, probably to ensure a certain stability. The greater the traction force applied, the greater the errors obtained with *M1* for the sagittal L5/S1 joint moments. This phenomenon was not replicated with *M2* and *M3*. Moreover, *M3* seemed less accurate at predicting the sagittal L5/S1 joint moments than *M2*, even though the input data used were equivalent to the gold standard for one of the two team members. This is because, since force platform data are used for one team member, errors introduced by both team members’ kinematics and BSIP estimation interfere with GRF prediction for the other team member.

The mean errors reported for method *M1* meant that, almost throughout the transfer, the moment was overestimated using the prediction method. This result is in accordance with those obtained by Barrett and Dennis [13], who indicated that the traction/compression force applied results in decreased back moments. For methods *M2* and *M3*, no overall tendency to underestimate or overestimate was noted, but there was probably a random error around the reference value.

For asymmetrical moments, the error level was lower than for sagittal moments. However, since the amplitudes were lower, relative errors were slightly higher. Moreover, since antero-posterior forces had little influence on asymmetrical moments (only on the axial component of the moment), errors for *M1* were smaller. Similarly, *M3* had larger errors due to the accumulation of errors for both team members.

For similar handling tasks performed individually, Muller et al. [18] reported RMSE of 18.7 Nm, 10.6 Nm, and 12.6 Nm for the sagittal, frontal and transverse axes, respectively. These values are like those obtained with *M2* and *M3*. Moreover, by comparing bottom-up and top-down approaches, several studies reported levels of uncertainty when estimating L5/S1 joint moments by using intrumented handles with force sensors and force platforms [32, 36, 37]. These uncertainties ranged between 4 Nm and 24 Nm for the sagittal axis, between 4 Nm and 20 Nm for the frontal axis and between 2 Nm and 15 Nm for the transverse axis. Using *M2* or *M3*, the errors are in the range of uncertainty, regardless of box characteristics, team member or team composition.

### General discussion

Team handling is commonly recommended to reduce the load during manual handling of heavy loads. Despite its benefits, some factors represent a risk for low back injuries and new methods should be developed to assess load among team members.

Based on the results of this paper, the use of only motion data (*M1*) did not allow an accurate estimate of L5/S1 joint moments. The lack of information on the traction/compression force applied by each team member could generate a significant error in the anterior direction and therefore in the L5/S1 joint moment estimation.

Adding the traction/compression force as an input (*M2*) is an improvement since it allows L5/S1 joint moments to be estimated with a relatively small error, comparable to the uncertainty values for this type of estimation. This method provides very encouraging results if the aim is to analyze realistic team handling tasks in a laboratory. Traction/compression force can be measured, for example, with strain gauges integrated into the box and positioned on its longitudinal axis. This instrumentation does not require the use of handles and does not limit handlers’ movements to an area defined by force platforms. Applying strain gauge measuring devices in the field could make it possible to study back efforts in a more natural handling situation although it would be technically complicated to do, given the work context and the loads to be moved. One viable method would be to estimate the traction/compression force and then predict GRF using method *M2*. Future work will be carried out to investigate this possibility.

Considering the traction/compression force in the form of force platforms under one of the team members is another interesting approach in a laboratory setting (*M3*). This makes it possible to estimate L5/S1 joint moments with a gold standard method for one team member and with a relatively small error for the other. When few force platforms are available, it is possible to assemble them to obtain a large displacement area for one team member. The other is then not constrained by any platform. Moreover, method *M3* does not require any instrumentation of the displaced load, which can be complex, for example when instrumenting a stretcher loaded with a patient.

The proposed methods can be used to analyze the load distribution between handlers in various work situations. The experimental conditions used in this paper show that it is possible to work with handlers of different genders, sizes and weights, and to analyze the influence of the carried load’s characteristics by considering, for example, off-center loads.

### Limits and perspectives

This work has several limitations. First, the tasks performed did not include foot displacements. This was constrained by the need to use force platforms. Other complementary studies involving foot displacements will have to be done, either by using several large platforms or by adding instrumented handles on the box. Nevertheless, this type of task has been used in the literature to validate prediction methods for handling tasks [18, 19]. Secondly, the traction/compression force was estimated from GRF and motion. These data were then considered as inputs for prediction method *M2*. It was reported that the estimation of hand forces using this type of method induces an error of about 20 N [29]. The influence of this error on the prediction method could be the object of a complementary study. Thirdly, the use of these prediction methods requires knowledge of different characteristic instants of a handling task, such as lifting and deposit, to separate the handling phases. Thus, the experimenters knew when a force was or was not applied to the handlers’ hands. Identification using a video camera might be the easiest way of doing this but requires time-consuming post-processing. To automate processing, identification methods based on handlers’ motion could be developed and implemented [18]. Fourthly, BSIP were extracted from anthropometric tables. Errors in BSIP estimation induce errors in GRF prediction [38] and in joint torque estimation [39, 40]. Improving the estimation of BSIP using calibration methods could improve the joint torque estimation, especially for subjects distant from the 50th percentile. Finally, to increase the realism of the team handling tasks to be analyzed, the ideal is to be able to perform experiments in the field in order to observe and analyze real work tasks. In this situation, the use of an optoelectronic motion capture system is no longer feasible. Previous studies have used the Lumbar Motion Monitor [2, 11] to measure low back kinematics in the field, but the proposed prediction methods require the whole body to be measured. An interesting extension of this current study would be to adapt prediction methods to wearable technologies such as magnetic and inertial measurement units [41–43], depth cameras [44, 45] or video cameras [46]. Since the accuracy of kinematic data plays a major role in prediction methods [38], studies must be carried out to verify the accuracy levels that can be obtained with this type of instrumentation.

## Conclusion

This study showed that the use of motion data alone does not allow for accurate estimation of back loading during team handling tasks. The traction/compression force applied by each handler does not affect motion but does influence back loading. Adding this force as an input allows for more accurate estimation. This force can be measured using strain gauges placed in the boxes or obtained indirectly using one force platform under one handler. Future work will also investigate the possibity of estimating this force to simplify the instrumentation. The prediction methods proposed here seem to be an interesting approach to analyze back loading during team handling in various work situations, with different team member anthropometric measurements and different box characteristics. Combining wearable motion capture systems with GRF prediction methods would allow one to perform this type of analysis in the field.

## Supporting information

S1 File (PDF)

S1 Data (CSV)

S2 Data (CSV)

S3 Data (CSV)
