# Supplementary material for: Back loading estimation during team handling: Is the use of only motion data sufficient?
Source: PLoS One. 2020 Dec 22;15(12):e0244405. doi: 10.1371/journal.pone.0244405 (PMC7755210; doi:10.1371/journal.pone.0244405)
Supplement: S1 File — (PDF) [file pone.0244405.s001.pdf]

Back loading estimation during team handling: is the use of only motion data sufficient?

S1 File

Marker positions

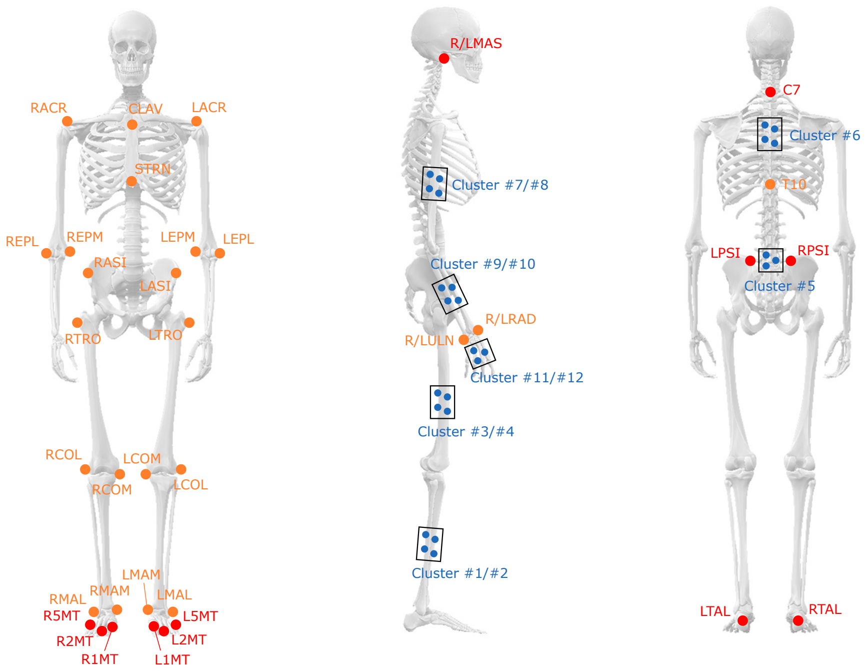

Figure 1: Locations of markers. Red markers were used in all motion captures; orange markers were only used during the calibration; blue markers constituted rigid clusters and were used in all motion captures to reconstruct the position of orange markers.

Table 1: Anatomical landmarks of markers.

R/LASI	Ilium anterior superior	T10	Spinous process of the 10th dorsal
R/LPSI	Ilium posterior superior	C7	Spinous process of the 7th cervical
Cluster ♯5	Pelvis	Cluster ♯6	Trunk
R/LTRO	Greater trochanter	STRN	Xiphoid process
Cluster ♯3/♯4	Thigh	CLAV	Sternum jugular incisure
R/LCOL	Femur lateral epicondyle	R/LMAS	Mastoid process
R/LCOM	Femur medial epicondyle	R/LACR	Acromion
Cluster ♯1/♯2	Leg	Cluster ♯7/♯8	Arm
R/LMAL	Lateral malleolus	R/LEPL	Lateral epicondyle of humerus
R/LMAM	Internal malleolus	R/LEPM	Medial epicondyle of humerus
R/L1MT	Head of 1st metatarsus	Cluster ♯9/♯10	Forearm
R/L2MT	Head of 2nd metatarsus	R/LULN	Ulna-styloid process
R/L5MT	Head of 5th metatarsus	R/LRAD	Radius-styloid process
R/LTAL	Calcaneum posterior tuberosity	Cluster ♯11/♯12	Hand

Statistical significance

Table 2: Statistical significance (main and interaction effects) of the two-way repeated measures ANOVA.

			Methods (M)	Box configurations (B)	M × B
GRF	V	r	0.12	0.52	0.39
		RMSE	0.28	0.91	0.069
		rRMSE	0.35	0.29	0.059
	AP	r	< 0.001	0.28	< 0.001
		RMSE	< 0.001	< 0.001	< 0.001
		rRMSE	< 0.001	< 0.001	< 0.001
	ML	r	0.49	0.96	0.11
		RMSE	0.13	0.011	< 0.001
		rRMSE	0.024	0.98	< 0.001
L5/S1 joint moments	S	r	0.074	0.48	0.39
		RMSE	< 0.001	< 0.001	< 0.001
		rRMSE	0.001	0.072	0.001
	A	r	0.53	0.026	0.459
		RMSE	0.008	0.015	0.104
		rRMSE	0.027	0.308	0.036

V: vertical; AP: antero-posterior; ML: medio-lateral; S: sagittal; A: asymmetrical.
